# Assessment of Ultra-Short Heart Variability Indices Derived by Smartphone Accelerometers for Stress Detection

**DOI:** 10.3390/s19173729

**Published:** 2019-08-28

**Authors:** Federica Landreani, Andrea Faini, Alba Martin-Yebra, Mattia Morri, Gianfranco Parati, Enrico Gianluca Caiani

**Affiliations:** 1 Dipartimento di Elettronica, Informazione e Bioingegneria, Politecnico di Milano, 20133 Milan, Italy; 2 Istituto Auxologico Italiano, IRCCS, Department of Cardiovascular Neural and Metabolic Sciences, S. Luca Hospital, 20149 Milan, Italy; 3 Department of Biomedical Engineering, Lund University, 22100 Lund, Sweden; 4 Department of Medicine and Surgery, Università di Milano-Bicocca, 20126 Milan, Italy; 5 Consiglio Nazionale delle Ricerche, Istituto di Elettronica e di Ingegneria dell’Informazione e delle Telecomunicazioni, 20133 Milan, Italy

**Keywords:** ballistocardiography, seismocardiography, ultra-short heart rate variability, stress evaluation, smartphone, accelerometers

## Abstract

Body acceleration due to heartbeat-induced reaction forces can be measured as mobile phone accelerometer (m-ACC) signals. Our aim was to test the feasibility of using m-ACC to detect changes induced by stress by ultra-short heart rate variability (USV) indices (standard deviation of normal-to-normal interval—SDNN and root mean square of successive differences—RMSSD). Sixteen healthy volunteers were recruited; m-ACC was recorded while in supine position, during spontaneous breathing at rest conditions (REST) and during one minute of mental stress (MS) induced by arithmetic serial subtraction task, simultaneous with conventional electrocardiogram (ECG). Beat occurrences were extracted from both ECG and m-ACC and used to compute USV indices using 60, 30 and 10 s durations, both for REST and MS. A feasibility of 93.8% in the beat-to-beat m-ACC heart rate series extraction was reached. In both ECG and m-ACC series, compared to REST, in MS the mean beat duration was reduced by 15% and RMSSD decreased by 38%. These results show that short term recordings (up to 10 s) of cardiac activity using smartphone’s accelerometers are able to capture the decrease in parasympathetic tone, in agreement with the induced stimulus.

## 1. Introduction

Technology developments and device miniaturization have opened the possibility for hand-held devices such as smartphones to be used for physiological data collection. Through their embedded tri-axial accelerometers, the mobile phone is sensitive enough to record the vibrations generated by the beating heart, as accelerometer signal (m-ACC) of milligravity (mg) level. In this way, the movements along the lateral, the normal, and the longitudinal direction can be detected.

Depending on the accelerometers position, these signals usually resemble:

(1) The ballistocardiogram (BCG), measuring the displacement of the mass of ejected blood from the ventricles through the aorta and then towards the peripheral circulation, represented by a series of systolic (I, J, K) waves describing the forces associated to the shifting of the center of body mass [1,2,3];
(2) The seismocardiogram (SCG), capturing the sequence of mechanical cardiac events known as isovolumetric contraction (IVC), aortic valve opening (AO), and aortic valve closure (AC) relevant to the systolic period [3,4,5,6].

The heartbeat fiducial points on the m-ACC signal associated to the sharp cardiac vibration waves in concomitance to the systolic activity can be detected using an electrocardiogram (ECG)-independent processing algorithm, and used to compute the cardiac interbeat interval, thus obtaining corresponding beat-to-beat time series. The feasibility and accuracy of measuring the beat-to-beat heart rate using smartphone accelerometers has been recently demonstrated [7,8,9,10,11,12,13].

Heart rate variability (HRV) analysis is usually applied to the series of time intervals between consecutive R-wave peaks (RR) extracted from the ECG, thus providing quantitative markers to evaluate the influence of the autonomic nervous system (ANS) on the heart rate [14,15,16,17]. The HRV approach considers monitoring periods that may range from 5 min up to 24 h [15], providing information that may be related to physiological status such as diabetic neuropathy [17,18], myocardial dysfunction [19], or stress conditions [20]. The feasibility of applying HRV analysis to beat-to-beat series obtained by accelerometric recordings of 5 minutes length has been already proven [12].

In the context of self-monitoring individual’s health and well-being status, the interest in using shorter recordings (<5 min) in stationary conditions of real-life scenarios suitable for HRV analysis is emerging, thus increasing user compliance and reliability of measurement. To this purpose, the ultra-short heart rate variability (USV) time domain indices—the standard deviation of normal-to-normal intervals (SDNNs) and the root mean square of successive differences (RMSSD)—have been proposed as a surrogate to assess the ANS influence on the heart rate [21,22,23,24].

We hypothesized that the USV analysis could be applied to assess the level of stress from the accelerometric signals acquired for short periods using a mobile phone, thus facilitating this self-assessment procedure without the need of other wearables or sensors, and overcoming the main limitation of keeping in position the device for longer periods.

Accordingly, our aim was to test the feasibility of detecting changes in the ANS state provoked by a mental task by using the beat-to-beat series from short recordings (<1 min) extracted by a mobile phone m-ACC signal. To attain that objective the USV indices were computed and compared with the indices obtained by the conventional ECG-RR series, considered the gold standard, simultaneously extracted. In addition, the ability to detect these changes using sub-segments of shorter durations (up to 10 s) was explored.

## 2. Materials and Methods

### 2.1. Study Population

A total of 16 subjects (age range 19–28, six females) were recruited, whose anthropometrics data are reported in Table 1. The experimental procedures described in this paper agreed with the ethics defined in the Helsinki Declaration of 1975, as revised in 2000. Each subject also provided voluntary written, informed consent to participate in the experimental protocol approved by the Ethical Committee of the Ospedale San Luca in Milan.

### 2.2. Accelerometric Signal Acquisition 

**Figure 1:**
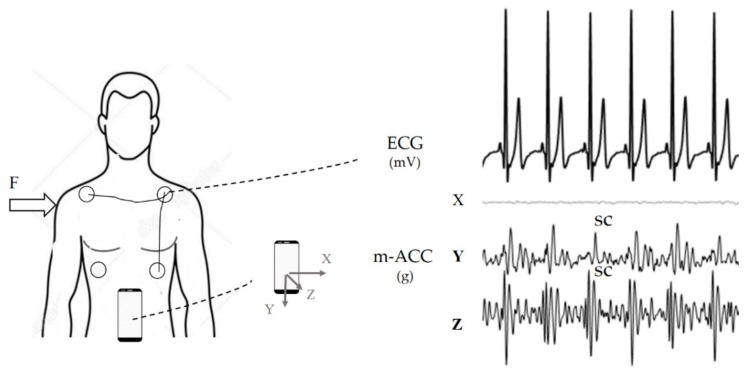
Theelectrodes and a smartphone were positioned on the subject: the electrocardiogram (ECG) signals were acquired simultaneously and synchronized by a motion artifact caused by an impulsive force (F) impressed on the subject’s shoulder. The ECG and the simultaneously acquired mobile phone tri-axial accelerometric signals (m-ACC) are shown: While the lateral (X) component does not project any heartbeat vibration, the longitudinal (Y) and normal (Z) components show a clear periodic complex (SC) related to cardiac heartbeat activity.

Each volunteer was studied in supine position using a smartphone (iPhone 6s, Apple Inc., Cupertino, CA, United States), positioned directly on the navel, with the phone top towards the head (Figure 1). The 3-orthogonal axis accelerometric signals (m-ACC, fs = 100 Hz, accelerometer sensitivity of 0.001 g) were acquired using the app ‘SensorLog’ v.2.4, resulting in three oriented channels corresponding to lateral (X), longitudinal (Y), and normal (Z) directions, simultaneously with a 6-leads electrocardiogram (ECG, Nexfin HD monitor, BMEYE, Amsterdam; fs = 1000 Hz). Despite the morphology of the m-ACC signal depends on the device position on the subject’s body [9], Y and Z components showed a major informative content relevant to heartbeat occurrence. For this reason, they were chosen to be processed with the ECG-free heartbeat detection.

**Figure 2:**
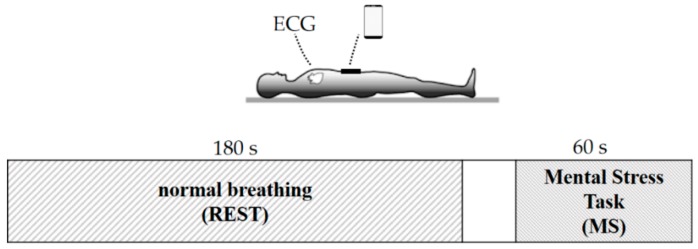
Schematic representation of the experimental protocol, where the subject was acquired in supine posture with a smartphone positioned on the belly above the navel. The protocol consisted in two steps: Normal breathing (REST) and the mental stress task (MS) where the subject was asked to perform arithmetic serial subtractions starting from a 4-digit number.

The signals were synchronized by applying a lateral impulsive force stimulus applied on to the subject’s shoulder, which was detected both by the smartphone’s accelerometers and by the ECG electrodes (as a movement artifact). After a 10 min acclimation period in supine posture, the experimental protocol included two acquisitions performed sequentially (see Figure 2): The former, lasting 3 minutes with the subject breathing normally in resting conditions (REST) and, after 1 minute of readjustment, the latter lasting 1 minute with a mental stress (MS) condition. As mental arithmetic is one of the most commonly utilized laboratory psychological stressors able to increase heart rate (HR) [25,26,27], stress was provoked as follows: The subject was instructed before the beginning of the experiment on how this phase would have been performed: Given a starting 4-digit number told to the subject at the beginning of the mental stress acquisition, the subject had to perform silently arithmetic serial subtractions of seven from that number at a pace of one every ten seconds, and communicate the final result only at the end of the 1-min acquisition. Preventing subjects speaking during the acquisition was a design choice, as communication between the subject and the researchers would have hindered the signal quality, as the acquired signal is sensitive to vibrations that are generated by speaking. 

### 2.3. Signal Processing

#### 2.3.1. Pre-Processing

To synchronize the smartphone signals with the ECG, the spike motion artifact introduced during the measurement was identified on the X component of the m-ACC signal and on the ECG lead I. After this step, the Y and Z components of the m-ACC signal were both band-pass filtered (4th order Butterworth filter): Cut-off frequencies of 5 and 25 Hz were used for Z, while 1 and 30 Hz for Y. This approach removed the out-of-band noise and breathing activity-related motion artifacts. The chest wall vibrations and the body acceleration due to the heartbeat-induced recoil forces were thus retained considering these different band-pass ranges [8].

#### 2.3.2. Heartbeat Detection and Algorithm Performance

On the ECG signal, R peaks were detected using Pan-Tompkins algorithm [28,29] and used to derive the RR series for comparison purposes with smartphone-derived series.

**Figure 3:**
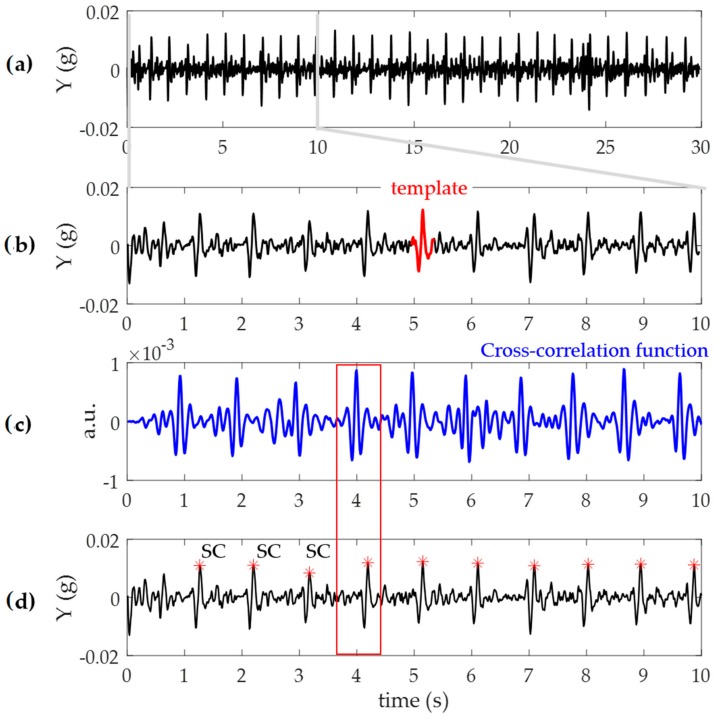
Schematic representation of the ECG-free heartbeat detection algorithm. (**a**): A 30 s segment of the m-ACC signal recording (black); (**b**) within the first 10 s segment, a template (red) is automatically selected; (**c**) the cross-correlation function (in blue) was obtained using the template with the 30 s signal (black) and the position of maximum values of cross-correlation were used to identify a search window (in red, minus sign) for each heartbeat; and (**d**) the windows were thus used to detect the systolic complex (SC, red dots) on the m-ACC signal (black).

On both Y and Z components of the m-ACC signal, to detect the systolic complex (SC), an ECG-free algorithm based on template matching technique was applied [30], performing the following steps:

- Signals were divided into 30 s segments (Figure 3a);
- For each segment, centered at the absolute maximum within the first 10 s, a template of 400 ms duration was extracted (Figure 3b): Such duration was chosen keeping into consideration the possible minimum duration of the heart cycle interval in case of a fast heart rate (~150 bpm);
- Computation of the cross-correlation between the identified template and the 30 s signal segment (Figure 3c);
- Searching windows centered at each maxima of the cross-correlation function (Figure 3c), dashed red) were defined and used to precisely locate each SC (red dot) as the wave with the maximum absolute amplitude on the m-ACC signal (black; Figure 3d). The length of the search window was calculated taking into consideration the mean of the previous three heartbeats durations.

**Figure 4:**
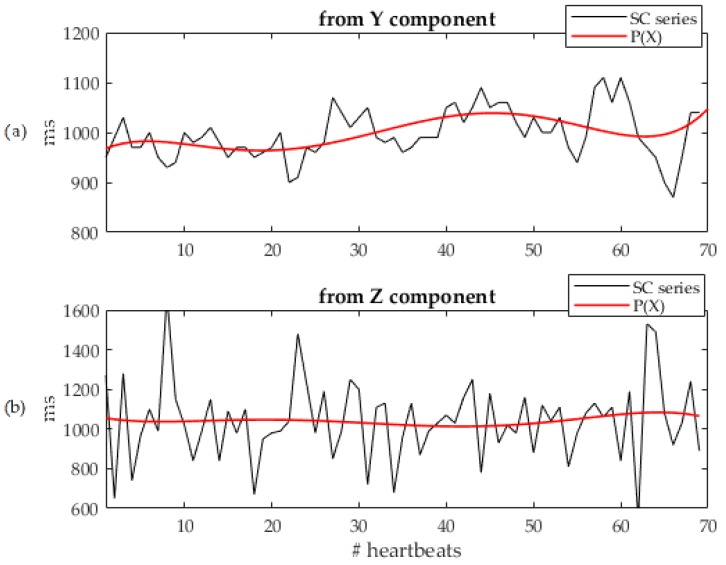
Polynomial interpolation series P(X) (in red) over imposed to the corresponding systolic complex beat-by-beat duration series (SCs; in black), separately for Y (**a**) and Z (**b**) acceleration components. See text for more details.

Then, beat-to-beat duration SC series were calculated as the distance between two consecutive SCs, for both Y and Z components. Then, the algorithm automatically selected the optimal (OPT) component as follows (see Figure 4):

- Least-squares fitting with a 5th order polynomial P(X) of the calculated SCs series for the Y and Z components (see Figure 4a,b, respectively);
- For each component, computation of the mean distance value between the SCs and the P(X) corresponding value;
- The component with the lowest distance was selected for further processing.

This step allowed us to automatically select the series with fewer outliers in presence of possible artifacts and missed or wrong detections, thus obtaining at least a beat-to-beat series for each subject and condition, from which to proceed with the USV indices computation.

#### 2.3.3. Ultra-Short Heart Rate Variability Indices 

The SDNN and RMSSD were computed as USV time domain indices. The SDNN estimates overall HRV, while RMSSD is actually an estimate of high frequency variation in HR led by the vagal tone activity of the ANS [15,21,31,32]. They were calculated using the most central 60 s, 30 s, and 10 s signal segments of the REST and MS recordings, from both RR and OPT series. In addition, to evaluate the possible implications of different selection criteria, the USV indices were computed also for the initial and final (30 s and 10 s length) segments on the captured series.

### 2.4. Statistical Analysis

Results are presented as medians (25th–75th percentile). 

The algorithm’s feasibility was computed as the number of acquisitions in which at least one optimal series for the ultra-short heart rate variability analysis was obtained, with respect to the total number of acquisitions. The sensitivity was calculated as the percentage ratio between the SC detected with respect to the corresponding R-wave ECG peaks. To evaluate the accuracy of the applied ECG-free detection algorithm, all the peaks were visually inspected together with ECG annotations: The misdetections, as double detection or incorrect peaks, were identified and categorized as false positive (FP), while the missing detections were considered as false negative (FN), where the true positive (TP) corresponded to a detection in the correct position (see Equation (1)).

(1) $$\mathrm{accuracy}=\frac{\mathrm{TP}}{\mathrm{FP}+\mathrm{FN}+\mathrm{TP}}.$$

In order to test if the OPT series could represent a valid surrogate for electrodes-free heart beat duration extraction, linear correlation and Bland-Altman analyses were computed by comparison with the corresponding RR series.

A Mann-Whitney unpaired test (*: *p* < 0.05) was applied to compare the OPT versus RR series parameters (cardiac cycle median duration and USV indices) to support the hypothesis that the obtained parameters represent the same information, both in REST and MS. 

Friedman test and multi-comparison Bonferroni correction were applied to test whether heartbeat mean duration and USV indices obtained from different length recordings (60 s, 30 s, and 10 s) were representative of the same distribution (#: *p* < 0.05), to support the hypothesis of using the shortest possible acquisition length, and to evaluate the effect of the segment position (initial, middle, and final) on the obtained results (†: *p* < 0.05).

A non-parametric Wilcoxon paired test (*: *p* < 0.05) was used to test significant differences in ANS sympatho-vagal status evidenced by the USV parameters between REST and MS, separately for the ECG and OPT series.

## 3. Results

### 3.1. Algorithm Perfomance

The m-ACC signals from one subject in REST and one in MS were discarded due to poor signal-to-noise ratio, thus resulting in a feasibility of the beat-to-beat heart rate series extraction of 93.8%. The algorithm automatically selected the Z component as the best component for heartbeat detection in 10/15 subjects for REST and in 11/15 subjects for MS.

Compared to the 1784 R-ECG peaks in REST and 1271 in MS, the ECG-free detection algorithm detected correctly 1754 beats in REST and 1246 beats in MS, thus resulting in an algorithm sensitivity of 98.3% and 98%, respectively, with high accuracy (98%) reached.

In addition, due the presence of ectopics beats in both REST and MS OPT series that could result in erroneous USV indices, another subject was discarded. Accordingly, to allow a paired comparison, 13 subjects were considered for further analysis.

### 3.2. Cardiac Cycle Duration

**Figure 5:**
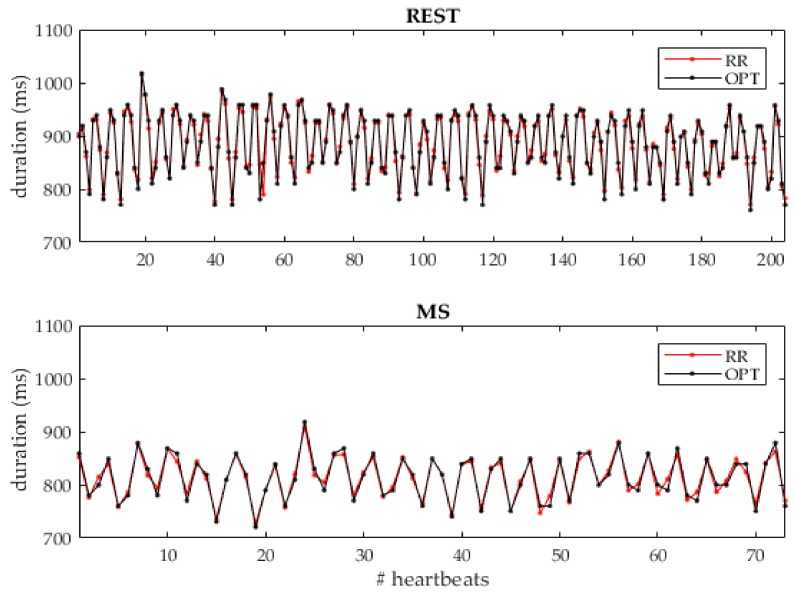
Example of the beat-to-beat RR series (red) and optimal (OPT; black) heart cycle duration series obtained at REST and MS for a representative subject. At REST, the measurement lasts 180 s, while the MS lasts 60 s.

In Figure 5, an example of the RR and OPT beat-to-beat variability series derived from a representative subject at REST and during MS are shown superimposed: It is possible to appreciate in both conditions the correspondence of the measured heart cycle durations.

**Figure 6:**
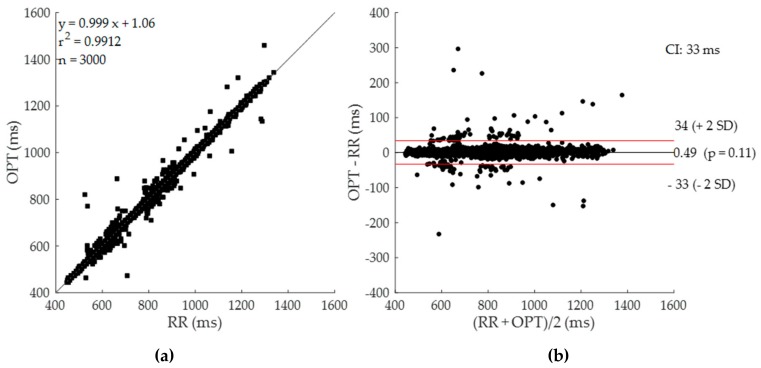
Linear correlation (**a**) and Bland-Altman (**b**) analyses obtained considering all the series (RR and OPT) obtained at REST and MS for a total of 3000 heartbeats. R^2^ coefficient and confidence’s intervals (CI = ±2 SD) are shown.

In Figure 6, the result of linear correlation and Bland-Altman analyses by comparing the gold standard RR versus the corresponding beat-by-beat OPT series are presented. It is possible to appreciate the high r^2^ value (0.99) and the narrow confidence interval (CI = ±33 ms, ±2 SD) achieved by the proposed method, tested globally in a range of heartbeat duration from 447 ms up to 1337 ms.

**Figure 7:**
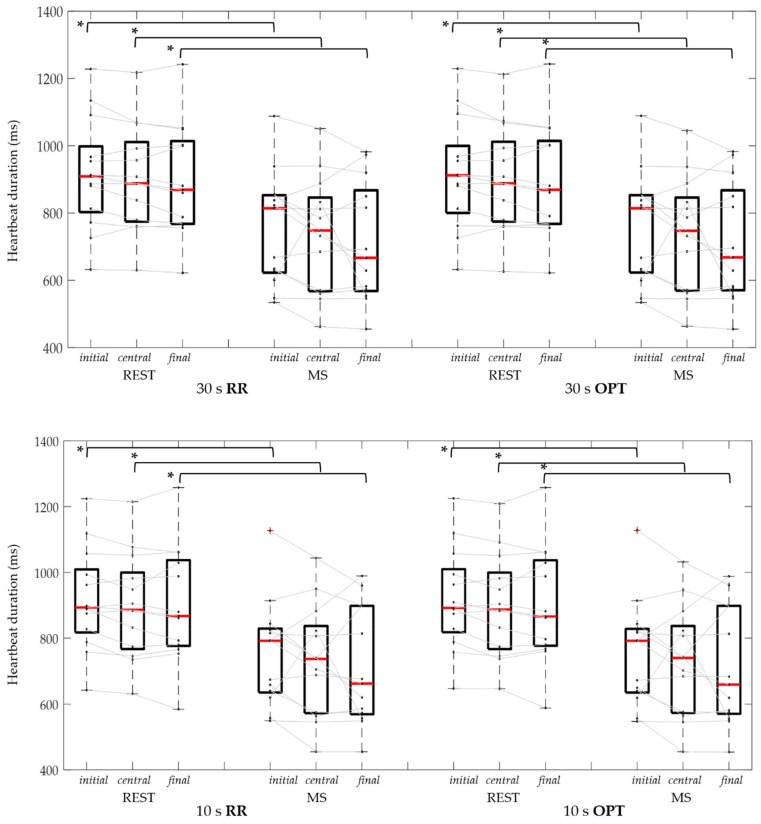
Heart beat duration distributions and individual data of RR and OPT series, in control (REST) and mental stress (MS) conditions, using 30 s (upper panel) and 10 s (bottom panel) signal segments considered at initial, central, and final position on the considered series. *: *p* < 0.05 REST vs. MS (Wilcoxon test).

In Table 2 and Figure 7, the results relevant to the heart cycle duration obtained considering different durations and segment positions on the ECG (RR) and the optimal (OPT) series by the smartphone’s accelerometer are presented. No statistical differences (Mann-Whitney and Friedman test) were found between RR and OPT for each duration and position on captured series, in both conditions. Changes induced by the stress condition were visible independently from the duration and segment position in both RR and OPT. In particular, for a 60 s duration, both the RR and OPT were found reduced by 16% (10%–26%); by considering the most central segment the RR and OPT were reduced by 17% (10%–27%) for 30 s duration, while using 10 s duration the RR and OPT were reduced by 20% (9%–27%). Interestingly, a trend of decrease in heartbeat duration during MS was visible within the 60 s period.

### 3.3. Ultra-Short Heart Rate Variability Indices

**Figure 8:**
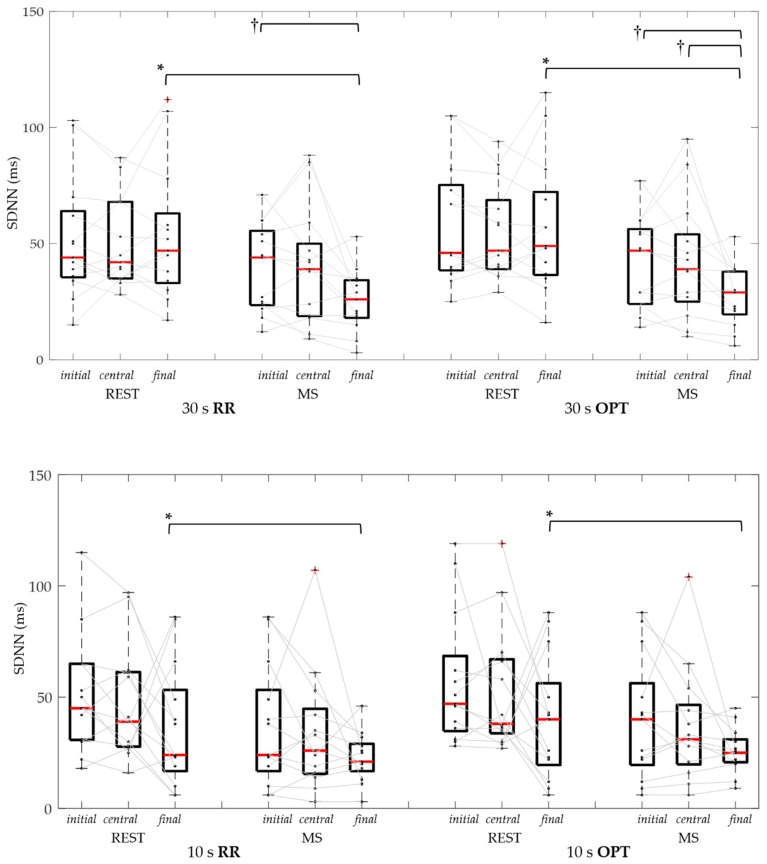
Standard deviation of normal-to-normal interval (SDNN) distributions and individual data of RR and OPT series, in control (REST) and mental stress (MS) conditions, using 30 s (upper panel) and 10 s (bottom panel) signal segments considered at the initial, central, and final series portion. *: *p* < 0.05 REST vs. MS (Wilcoxon test); †: *p* < 0.016 among segment positions (Friedman test and Bonferroni correction).

**Figure 9:**
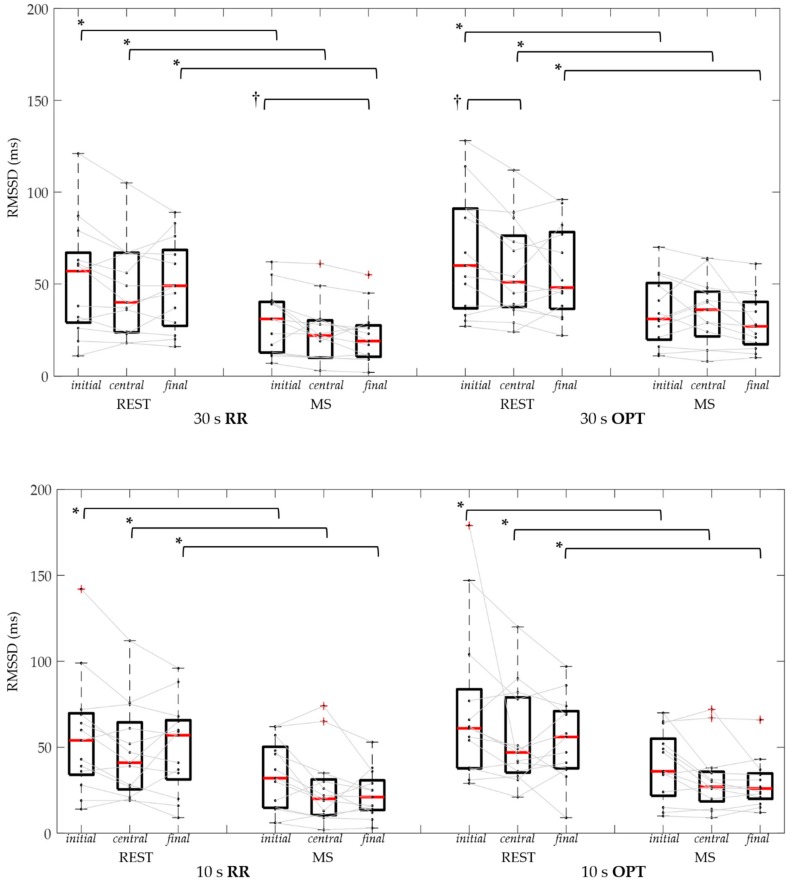
Root mean square of successive differences (RMSSD) distributions and individual data of RR and OPT series, in control (REST) and mental stress (MS) conditions, using 30 s (upper panel) and 10 s (bottom panel) signal segments considered at initial, central, and final series portion. *: *p* < 0.05 REST vs. MS (Wilcoxon test); †: *p* < 0.016 vs. initial portion (Friedman test and Bonferroni correction).

In Table 3 and Figure 8, and Table 4 and Figure 9, the results of USV indices (SDNN and RMSSD, respectively) obtained considering different durations and positions of RR and OPT series are reported. It is possible to notice that results obtained from RR and OPT series were very similar, in each tested condition.

Regarding SDNN, no statistical difference was found between REST and MS, both using ECG and OPT, when considering the whole 60 s duration, or the initial and central portions of the shorter durations. On the contrary, the SDNN was significantly reduced from CTRL to MS, for both RR and OPT series, in the final 30 s and 10 s segments, previously associated to shortened mean heart duration. 

The Friedman test showed that SDNN had values proportional with the duration of the RR and OPT series during MS, with statistical significance when comparing the 60 s with the central 30 s and 10 s indices.

Regarding RMSSD, independently from the duration or the position of the segment, the mental stress condition resulted in reduced values of the computed parameter compared to REST for both RR and OPT series. In particular, for the 60 s segment the RMSSD in RR was reduced by 38% (26%–71%), and by 40% (8%–68%) in OPT. Considering the central 30 s segment, it was reduced by 45% (27%–72%) in RR, and by 46% (22%–64%) in OPT, while for the central 10 s segments it was reduced by 53% (13%–73%) in RR, and by 49% (8%–66%) in OPT.

## 4. Discussion

Current smartphone technology and embedded sensors have the potential to acquire signals related to cardiac activity. In addition to the use of the on-board camera to derive the pulsation of the skin capillary blood flow in the fingertips from which to obtain the pulse rate [33], micro-electro-mechanical systems technology embedded in smartphones potentially allows measuring heart mechanical activity by acquiring vibrational signals when positioned on the body [9]. The potential of these approaches in using the smartphone as a source of vital parameters stands in the ability to acquire these signals anytime and anywhere, without the need of additional peripherals, thus improving patient empowerment through self-measurement.

However, in order to be accepted by the medical community, the potential value relevant to the use of these technologies needs to be proved. While the use of smartphone cameras using photoplethysmography for early detection of atrial fibrillation, based on the analysis of beat-by-beat duration variability series, has initially proved its value in a prospective two-center, international clinical validation study [33], the validation of using smartphone accelerometers is still limited and again focusing on beat-by-beat duration variability only [8,10,34].

Our hypothesis was that the beat-to-beat heart rate series derived by a smartphone without any peripheral were suitable for the USV analysis, potentially useful for stress evaluation from a short time series. To this purpose, the feasibility of detecting changes in the ANS sympatho-vagal state provoked by a mental task in normal volunteers was tested.

The choice of acquiring the accelerometric signal from the navel with the subject in the supine position was guided from previous results we obtained in a preliminary analysis [8], in which the feasibility of acquisitions and accuracy of the beat-by-beat measurements were tested with the smartphone positioned on the thorax around the cardiac apex position and on the navel, with subjects in the supine and standing posture. With the aim of including both male and females volunteers in this study, thus overcoming possible gender-related limitations with the thoracic position, and in the perspective of a real applicability scenario including daily self-assessment in patients performed in supine resting condition in the morning, when clinical guidance suggest to obtain these kind of measurement [35,36], the navel position was chosen as it guarantees easy reproducibility and is not influenced by gender-related body morphology. Moreover, in this position the smartphone does not require any external accessory to be worn.

In our work, the smartphone was used as a sensor for accelerometric data acquisition, showing a good feasibility (93.8%). Signal quality allowed further analysis, including automatic parameters extraction at least in one component of the smartphone tri-axial accelerometers. Acceptable limits of agreement corresponding to ±10 bpm for the fastest heart rate analyzed (134 bpm) and to ±1 bpm for the lowest one (45 bpm) were obtained, in agreement with previous studies [8,30,37]. 

The different durations of considered signal segments were selected as a compromise, taking into consideration an acquisition as short as possible in a hypothetical user-driven scenario, but at the same time being able to record a reasonable amount of data to provide reliable measurements. As already stated, for mobile applications short-term measurements are desirable for USV analysis, since the conventional five minute long recordings might be inadequately long and prone to artifacts.

The results of this study are in agreement with our preliminary findings [30] obtained in only six subjects, thus confirming the feasibility of applying USV to SC beat-to-beat measurements derived by the smartphone accelerometers. From the obtained results, we showed that the median heart rate could be accurately estimated from very short segments (even from 10 s acquisition) of m-ACC signals, without differences when compared to the ECG results. Through the mental stress task, the median heartbeat duration was found significantly shortened when compared to the rest condition, as physiological expected, and in line with what observed using the ECG derived series.

For both RR and OPT series, the obtained results for USV parameters showed a decrease in RMSSD induced by the stress condition visible for each signal duration and independently from the criterion used to choose the segment position. In SDNN, this was visible only when considering the last portion of the signal, for both 30 s and 10 s durations. As mental stress is known to increase sympathetic activity, as revealed by the increased heart rate and reduced SDNN, the mental exercise induced a significant decrease in parasympathetic activation, in agreement with the induced stimulus, reflected by the significant decrease in RMSSD in both ECG and OPT series. These results highlight the potential to use the smartphone’s accelerometers to derive cardiac beat-to-beat measurements, able to monitor a stress-induced situation as a decrease from a baseline value, using very short acquisitions (even from 10 s).

Once confirmed in a larger number of subjects, a 10 s or 30 s acquisition could be considered as an easy, non-invasive way for the self-evaluation of stress using accelerometers already embedded in the mobile phone. This technology could have potential benefits in both cardiac disease prevention and self-assessment of patients with chronic disease (such as diabetes [17,38], or in patients where an imbalance of cardiac autonomic activity plays an important role, such as in coronary heart disease [39,40,41,42]), where simple but effective monitoring tools are needed in order to have reliable at-home measurements, managed directly by the patient.

A possible limitation of this study is constituted by the low sampling rate (100 Hz) of the accelerometric signal obtained from the mobile phone, which could reflect a lower precision in the computed USV parameters. However, as visible from Table 3 and Table 4, the reported changes in values of SDNN and RMSSD between REST and MS in OPT series were very similar to those obtained by the ECG sampled at 1000 Hz, but with a distribution of values in REST and MS, both for SDNN and RMSSD, that was always higher than those of the ECG, related to the lower sampling rate. In addition, previous studies [43] showed that a 100 Hz sampling frequency could be considered still appropriate to produce acceptable results for time-domain heart-variability analysis (but not for frequency domain parameters).

## 5. Conclusions

The beat-to-beat heart rate variability series derived by a smartphone’s accelerometers were able to detect the changes in ultra-short term HRV indices relevant to a change in the sympatho-vagal balance activation, induced by a stressor stimulus. In particular, the USV feature of RMSSD obtained by the m-ACC signal of up to a 10 sec duration could be used as a potential marker to estimate the stress level compared to a control value. This simple approach and its potential application in stress evaluation generate a new value of using the embedded smartphone accelerometers as a new tool for self-tracking cardiac activity.

## Figures and Tables

**Table 1:** Anthropometric characteristics of the population in terms of age, weight, height, and body mass index (BMI) expressed as the median (25th–75th percentiles).

Age (y)	Weight (Kg)	Height (m)	BMI (Kg/m^2^)
22 (21–23)	67.5 (61–75.5)	180 (169–184)	21.5 (20.3–22.9)

**Table 2:** Heart beat duration (ms) obtained from the ECG (RR) and the optimal (OPT) series by the smartphone’s accelerometer expressed as a median (25th–75th percentiles), for 60 s, 30 s, and 10 s duration and different segments (initial, central, final), in control (REST) and mental stress (MS) conditions. *: *p* < 0.05 REST vs. MS (Wilcoxon test).

Heart Cycle Duration (ms)						
	REST	MS	REST	MS	REST	MS
	**60 ‘’**					
**RR**			887 (780–985)	740 * (580–827)		
**OPT**			887 (780–987)	741 * (584–830)		
	**30 ″**					
	**initial**		**central**		**final**	
**RR**	909 (813-967)	814 * (630–852)	888 (779–992)	748 * (570–832)	869 (769–1002)	667 * (573–850)
**OPT**	912 (813–968)	814 * (631–852)	888 (779–993)	747 * (571–832)	869 (768–1002)	668 * (576–850)
	**10 ″**					
**RR**	893 (827–993)	792 * (640–824)	886 (774–982)	737 * (575–822)	867 (780–1029)	662 * (573–898)
**OPT**	892 (828–924)	792 * (640–823)	887 (774–982)	740 * (576–822)	866 (780–1029)	659 * (575–898)

**Table 3:** SDNN values (ms) obtained from the ultra-short heart rate variability analysis using RR and optimal (OPT) series. SDNN are expressed as medians (25th–75th percentiles), for 60 s, 30 s, and 10 s signal segments duration and different segments (initial, central, and final), both in control (REST) and mental stress (MS) conditions. *: *p* < 0.05 REST vs. MS (Wilcoxon test); #: *p* < 0.016 vs. 60 s and †: *p* < 0.016 between segment position (Friedman test and Bonferroni correction).

	SDNN (ms)					
	REST	MS	REST	MS	REST	MS
			60 ″			
**RR**			48 (41–64)	42 (27–72)		
**OPT**			54 (43–66)	45 (32–72)		
	**30 ″**					
	**initial**		**central**		**final**	
**RR**	44 (36–62)	44 † (24–54)	42 (35–68)	39 #, † (28–61)	47 (34–58)	26 *, † (19–34)
**OPT**	46 (39–73)	47 † (24–55)	47 (40–65)	39 #, † (27–51)	49 (37–69)	29 *, † (21–38)
	**10 ″**					
**RR**	45 (31–65)	24 (19–49)	39 (19–47)	26 # (16–42)	44 (29-78)	21 * (18–28)
**OPT**	47 (36–62)	40 (22–50)	38 (35–66)	31 # (21–44)	46 (33–79)	25 * (21–30)

**Table 4:** RMSSSD values (ms) obtained from the ultra-short heart rate variability analysis using RR and optimal (OPT) series. RMSSD are expressed as medians (25th–75th percentiles), for 60 s, 30 s, and 10 s (initial, central, and final) signal segments duration, both in control (REST) and mental stress (MS) conditions. *: *p* < 0.05 REST vs. MS (Wilcoxon test); #: *p* < 0.016 vs. 60 s (Friedman test and Bonferroni correction); †: *p* < 0.016 between segment position (Friedman test and Bonferroni correction).

	RMSSD (ms)						
	REST	MS	REST	MS	REST		MS
	60 ″						
**RR**			39 (30–65)	23 * (14–23)			
**OPT**			48 (38–76)	36 * (25–48)			
	**30 ″**						
	**initial**		**Central**			**final**	
**RR**	57 (30–63)	31 *† (13–40)	40 (24–67)	22 * (10–30)	49 (29–66)		19 *† (11–27)
**OPT**	60 † (38–91)	31 * (21–49)	51 † (38-73)	36 * (24-45)	48 # (38–77)		27 * (18–39)
	**10 ″**						
**RR**	54 (36–69)	32 * (15-48)	41 (27-61)	20 * (11–30)	57 (35-65)		21 * (14-29)
**OPT**	61 (38–77)	36 * (24–52)	47 (36–78)	27 * (20–35)	56 (38–70)		26 * (21–24)

## References

[1] Starr I., Rawson A.J., Schroeder H.A., Joseph N.R. (1939). Studies on the estimation of cardiac ouptut in man, and of abnormalities in cardiac function, from the heart’s recoil and the blood’s impacts; The ballistocardiogram. Am. J. Physiol-Legacy Content.

[2] Gubner R.S., Rodstein M., Ungerleider H.E. (1953). Ballistocardiography; An appraisal of technic, physiologic principles, and clinical value. Circulation.

[3] Inan O.T., Migeotte P.F., Park K.S., Etemadi M., Tavakolian K., Casanella R., Di Rienzo M. (2015). Ballistocardiography and seismocardiography: A review of recent advances. IEEE J-BHI.

[4] Salerno D.M., Zanetti J. (1991). Seismocardiography for monitoring changes in left ventricular function during ischemia. Chest.

[5] Zanetti J.M., Salerno D.M. Seismocardiography: A technique for recording precordial acceleration. Proceedings of the Fourth Annual IEEE Symposium on Computer-Based Medical Systems.

[6] Zanetti J.M., Tavakolian K. Seismocardiography: Past, present and future. Proceedings of the 35th Annual International Conference of the IEEE Engineering in Medicine and Biology Society (EMBC).

[7] Landreani F., Martin-Yebra A., Casellato C., Pavan E., Frigo C., Migeotte P.F., Caiani E.G. Feasibility study for beat-to-beat heart rate detection by smartphone’s accelerometers. Proceedings of the E-Health and Bioengineering Conference (EHB).

[8] Landreani F., Martin-Yebra A., Casellato C., Frigo C., Pavan E., Migeotte P.F., Caiani E.G. Beat-to-beat heart rate detection by smartphone’s accelerometers: Validation with ECG. Proceedings of the 38th Annual International Conference of the IEEE Engineering in Medicine and Biology Society (EMBC).

[9] Landreani F., Caiani E.G. (2017). Smartphone accelerometers for the detection of heart rate. Expert Rev. Med. Devic..

[10] Sieciński S., Kostka P., Gzik M., Tkacz E., Paszenda Z., Piętka E. (2018). Determining Heart Rate Beat-to-Beat from Smartphone Seismocardiograms: Preliminary Studies. Proceedings of the Innovations in Biomedical Engineering (IBE), Zabrze, Poland, 19–20 October 2017.

[11] Gavriel C., Parker K.H., Faisal A.A. Smartphone as an ultra-low cost medical tricorder for real-time cardiological measurements via ballistocardiography. Proceedings of the IEEE 12th International Conference on Wearable and Implantable Body Sensor Networks (BSN).

[12] Ramos-Castro J., Moreno J., Miranda-Vidal H., Garcia-Gonzalez M.A., Fernandez-Chimeno M., Rodas G., Capdevila L. Heart rate variability analysis using a seismocardiogram signal. Proceedings of the Annual International Conference of the IEEE Engineering in Medicine and Biology Society.

[13] Koivisto T., Lahdenoja O., Hurnanen T., Knuutila T., Vasankari T., Kiviniemi T., Pankaala M. Detecting atrial fibrillation via existing smartphones without any add-ons. Proceedings of the ESC Congress.

[14] Rodriguez J., Blaber A.P., Kneihsl M., Ruedl R., Green D.A., Broadbent J., Goswami N. (2017). Poststroke alterations in heart rate variability during orthostatic challenge. Medicine.

[15] (1996). Heart rate variability. Standards of measurement, physiological interpretation, and clinical use. Task Force of the European Society of Cardiology and the North American Society of Pacing and Electrophysiology. Eur. Heart J..

[16] Thayer J.F., Yamamoto S.S., Brosschot J.F. (2010). The relationship of autonomic imbalance, heart rate variability and cardiovascular disease risk factors. Int. J. Cardiol..

[17] Chessa M., Butera G., Lanza G.A., Bossone E., Delogu A., De Rosa G., Carminati M. (2002). Role of heart rate variability in the early diagnosis of diabetic autonomic neuropathy in children. Herz.

[18] Pagani M., Malfatto G., Pierini S., Casati R., Masu A.M., Poli M., Malliani A. (1988). Spectral analysis of heart rate variability in the assessment of autonomic diabetic neuropathy. J. Auton. Nervous Syst..

[19] Nolan J., Flapan A.D., Capewell S., MacDonald T.M., Neilson J.M., Ewing D.J. (1992). Decreased cardiac parasympathetic activity in chronic heart failure and its relation to left ventricular function. Br Heart J..

[20] Taelman J., Vandeput S., Spaepen A., Van Huffel S., Vander Sloten J., Verdonck P., Nyssen M., Haueisen J. (2009). Influence of Mental Stress on Heart Rate and Heart Rate Variability. Proceedings of the 4th European Conference of the International Federation for Medical and Biological Engineering (IFMBE).

[21] Munoz M.L., Van Roon A., Riese H., Thio C., Oostenbroek E., Westrik I., Snieder H. (2015). Validity of (Ultra-)Short recordings for heart rate variability measurements. PLoS ONE.

[22] Baek H.J., Cho C.-H., Cho J., Woo J.-M. (2015). Reliability of Ultra-Short-Term Analysis as a Surrogate of Standard 5-Min Analysis of Heart Rate Variability. Telemed. E-Health.

[23] Pecchia L., Castaldo R., Montesinos L., Melillo P. (2018). Are ultra-short heart rate variability features good surrogates of short-term ones? State-of-the-art review and recommendations. Heal. Tech. Lett..

[24] Castaldo R., Montesinos L., Melillo P., James C., Pecchia L. (2019). Ultra-short term HRV features as surrogates of short term HRV: A case study on mental stress detection in real life. BMC Med. Inform. Decis. Mak..

[25] Sloan R.P., Korten J.B., Myers M.M. (1991). Components of heart rate reactivity during mental arithmetic with and without speaking. Physiol. Behav..

[26] Brown T.G., Szabo A., Seraganian P. (1988). Physical versus psychological determinants of heart rate reactivity to mental arithmetic. Psychophysiology.

[27] Sharpley C.F., Kamen P., Galatsis M., Heppel R., Veivers C., Claus K. (2000). An examination of the relationship between resting heart rate variability and heart rate reactivity to a mental arithmetic stressor. Appl. Psychophysiol. Biofeedback.

[28] Pan J., Tompkins W.J. (1985). A Real-Time QRS Detection Algorithm. IEEE Trans. Biomed. Eng.

[29] Sedghamiz H. (2014). Matlab Implementation of Pan Tompkins ECG QRS Detector. https://www.researchgate.net/publication/313673153_Matlab_Implementation_of_Pan_Tompkins_ECG_QRS_detector..

[30] Landreani F., Morri M., Martin-Yebra A., Casellato C., Pavan E., Frigo C., Caiani E.G. Ultra-short-term heart rate variability analysis on accelerometric signals from mobile phone. Proceedings of the E-Health and Bioengineering Conference (EHB).

[31] Salahuddin L., Cho J., Jeong M.G., Kim D. Ultra short term analysis of heart rate variability for monitoring mental stress in mobile settings. Proceedings of the 29th Annual International Conference of the IEEE Engineering in Medicine and Biology Society.

[32] Elghozi J.-L., Julien C. (2007). Sympathetic control of short-term heart rate variability and its pharmacological modulation. Fundam. Clin. Pharmacol..

[33] Brasier N., Raichle C.J., Dörr M., Becke A., Nohturfft V., Weber S., Eckstein J. (2019). Detection of atrial fibrillation with a smartphone camera: First prospective, international, two-centre, clinical validation study (DETECT AF PRO). Europace.

[34] Mohamed R., Youssef M. (2017). HeartSense: Ubiquitous Accurate Multi-Modal Fusion-based Heart Rate Estimation Using Smartphones. Proceedings of the ACM on Interactive, Mobile, Wearable and Ubiquitous Technologies.

[35] Böhm M., Reil J.-C., Deedwania P., Kim J.B., Borer J.S. (2015). Resting heart rate: risk indicator and emerging risk factor in cardiovascular disease. Am. J. Medic..

[36] Hamill V., Ford I., Fox K., Böhm M., Borer J.S., Ferrari R., Swedberg K. (2015). Repeated heart rate measurement and cardiovascular outcomes in left ventricular systolic dysfunction. Am. J. Medic..

[37] Landreani F., Hossein A., Golier D., Caiani E., Van de Borne P., Rabineau J., Migeotte P.-F. Heartbeat detection using three-axial seismocardiogram acquired by mobile phone. Proceedings of the Computing in Cardiology Conference (CinC).

[38] Nussinovitch U., Cohen O., Kaminer K., Ilani J., Nussinovitch N. (2012). Evaluating reliability of ultra-short ECG indices of heart rate variability in diabetes mellitus patients. J. Diabetes Complicat..

[39] Stein P.K., Carney R.M., Freedland K.E., Skala J.A., Jaffe A.S., Kleiger R.E., Rottman J.N. (2000). Severe depression is associated with markedly reduced heart rate variability in patients with stable coronary heart disease. J. Psychosom. Res..

[40] La Rovere M.T., Pinna G.D., Maestri R., Mortara A., Capomolla S., Febo O., Cobelli F. (2003). Short-term heart rate variability strongly predicts sudden cardiac death in chronic heart failure patients. Circulation.

[41] Carney R.M., Saunders R.D., Freedland K.E., Stein P., Rich M.W., Jaffe A.S. (1995). Association of depression witk reduced heart rate variability in coronary artery disease. Am. J. Cardiol..

[42] Kupari M., Virolainen J., Koskinen P., Tikkanen M.J. (1993). Short-term heart rate variability and factors modifying the risk of coronary artery disease in a population sample. Am. J. Cardiol..

[43] Kwon O., Jeong J., Kim H.B., Kwon I.H., Park S.Y., Kim J.E., Choi Y. (2018). Electrocardiogram sampling frequency range acceptable for heart rate variability analysis. Healthc. Inform. Res..

